# Genomic characterization of SARS-CoV-2 in Egypt: insights into spike protein thermodynamic stability

**DOI:** 10.3389/fmicb.2023.1190133

**Published:** 2023-06-02

**Authors:** Deena Jalal, Omar Samir, Mariam G. Elzayat, Hend E. El-Shqanqery, Aya A. Diab, Lamiaa ElKaialy, Aya M. Mohammed, Donia Hamdy, Islam K. Matar, Khaled Amer, Mostafa Elnakib, Wael Hassan, Tarek Mansour, Sonia Soliman, Reem Hassan, Ghada M. Al-Toukhy, Mahmoud Hammad, Ibrahim Abdo, Ahmed A. Sayed

**Affiliations:** ^1^Department of Basic Research, Genomics and Epigenomics Program, Children’s Cancer Hospital Egypt 57357, Cairo, Egypt; ^2^Department of Chemistry, Saint Mary’s University, Halifax, NS, Canada; ^3^Egypt Center for Research and Regenerative Medicine (ECRRM), Cairo, Egypt; ^4^Department of Virology and Immunology, National Cancer Institute, Cairo University, Cairo, Egypt; ^5^Department of Clinical Pathology, Children’s Cancer Hospital Egypt 57357, Cairo, Egypt; ^6^Department of Clinical Pathology, National Cancer Institute, Cairo University, Cairo, Egypt; ^7^Department of Clinical and Chemical Pathology, Kasr Al-Aini School of Medicine, Cairo University, Cairo, Egypt; ^8^Molecular Microbiology Unit, Children’s Cancer Hospital Egypt 57357, Cairo, Egypt; ^9^Department of Virology and Immunology, Children’s Cancer Hospital Egypt 57357, Cairo, Egypt; ^10^Department of Pediatric Oncology, National Cancer Institute, Cairo University, Cairo, Egypt; ^11^Department of Pediatric Oncology, Children’s Cancer Hospital Egypt 57357, Cairo, Egypt; ^12^Department of Clinical Pharmacy, Children’s Cancer Hospital Egypt 57357, Cairo, Egypt; ^13^Faculty of Science, Department of Biochemistry, Ain Shams University, Cairo, Egypt

**Keywords:** SARS-CoV-2, Egypt, genome sequencing, phylogenetic analysis, mutations, spike, protein stability

## Abstract

The overall pattern of the SARS-CoV-2 pandemic so far has been a series of waves; surges in new cases followed by declines. The appearance of novel mutations and variants underlie the rises in infections, making surveillance of SARS-CoV-2 mutations and prediction of variant evolution of utmost importance. In this study, we sequenced 320 SARS-CoV-2 viral genomes isolated from patients from the outpatient COVID-19 clinic in the Children’s Cancer Hospital Egypt 57357 (CCHE 57357) and the Egypt Center for Research and Regenerative Medicine (ECRRM). The samples were collected between March and December 2021, covering the third and fourth waves of the pandemic. The third wave was found to be dominated by Nextclade 20D in our samples, with a small number of alpha variants. The delta variant was found to dominate the fourth wave samples, with the appearance of omicron variants late in 2021. Phylogenetic analysis reveals that the omicron variants are closest genetically to early pandemic variants. Mutation analysis shows SNPs, stop codon mutation gain, and deletion/insertion mutations, with distinct patterns of mutations governed by Nextclade or WHO variant. Finally, we observed a large number of highly correlated mutations, and some negatively correlated mutations, and identified a general inclination toward mutations that lead to enhanced thermodynamic stability of the spike protein. Overall, this study contributes genetic and phylogenetic data, as well as provides insights into SARS-CoV-2 viral evolution that may eventually help in the prediction of evolving mutations for better vaccine development and drug targets.

## Introduction

SARS-CoV-2 belongs to the family of betacoronaviruses, and like other RNA viruses, it is characterized by their high mutation rates (Duffy, 2018). This is attributed to their small genome size, the use of RNA polymerases as a replicase which lacks proof-reading activity, and complicated by the fact that they encode their own replicase, and thus can introduce mutations in their RNA dependent RNA polymerase, RdRp, favoring the introduction of further mutations to improve their competitive fitness (Duffy, 2018). Other RNA viruses such as coronaviruses (MERS and SARS-CoV), influenza viruses, and polio viruses have all exploited this for crossing the species barrier, re-infection, evading vaccine-induced immune response, and prevailing for extended periods of time.

Following the initial onset of SARS-CoV-2, declines in number of cases followed by surges of COVID-19 infections created a wave pattern for the pandemic. The rises in COVID-19 infection rates primarily occur due to the appearance of new variants resulting from mutations in the viral genome (Dutta, 2022; Lin et al., 2022). The WHO denotes some variants as variants of interest (VOIs) and variants of concern (VOCs) according to the associated risk and spread. After an initial decline in number of cases in Summer 2020, a more aggressive second wave of COVID-19 occurred during Winter 2020–2021, with the initial appearance of the first VOC, the alpha variant, in the United Kingdom. The second wave occurred concomitant with lifting of nation-wide lockdowns, reduced adherence with health guidelines and followed the natural periodicity frequently observed in viral infections. The arrival of FDA-authorized vaccines helped curb the intensity of the second wave, but was soon followed by a third wave after the spread of the alpha variant worldwide. A short-lived reduction in cases following world-wide vaccination efforts was hindered by the appearance of the delta variant in the fourth wave, and later the omicron variant that still persists worldwide until this day. The omicron variant is the current predominant variant and has caused fifth and sixth waves of the pandemic. The first reported case of SARS-CoV-2 in Egypt was in February 2020, and a nation-wide curfew was initiated in March 2020. As of March 2023, the total confirmed COVID-19 cases are estimated to be 515,698, and deaths to be 24,809, with a case-fatality rate of 4.81% [Coronavirus Pandemic (COVID-19), n.d.]. Due to limited testing in Egypt, however, the actual numbers are expected to be much higher.

Due to the importance of spike protein in viral cell entry, infectivity and antibody recognition, it is considered the most important concern in SARS-CoV-2 evolution. Many non-synonymous mutations in the spike protein appear in the VOCs identified in addition to D614G which became a predominant spike mutation since the first wave of COVID-19. The spike mutations underlie the higher transmissibility and spread observed in these VOCs. The alpha variant (Nextclade 20I, PANGO B.1.1.7), shows many non-synonymous mutations that are of immunological importance (Lippi et al., 2021). The deletion at positions 69 and 70 (Δ69-70) has been associated with failure of diagnostic tests, and an increased infectivity of the virion (Lippi et al., 2021; Volz et al., 2021). Similarly, the N501Y mutation was shown to increase ACE2 binding and consequently cell infectivity (Luan et al., 2021; Tian et al., 2021). The delta variant (Nextclades 21A, I and J, B.1.617.2), on the other hand, harbored several other spike mutations including two which occur in the RBD domain, L452R and T478K. The latest identified VOC, the omicron (Nextclade 21K, PANGO B.1.1.529), is highly mutated containing more than 50 mutations throughout its genome among which at least 32 mutations are in the spike glycoprotein. Some of the spike mutations in the omicron variant overlap with other VOCs such as T478K and N501Y (Callaway, 2021; Bhattacharya et al., 2022; Gao et al., 2022), whereas the rest are unique to the omicron variant. Functional aspects of the spike protein all depend on the protein structure, and overall folding stability is a major selection pressure on the evolution of new mutations (Tokuriki et al., 2008; Liberles et al., 2012). Development of new mutations enhancing ACE2 binding or vaccine evasion are less likely if the spike loses its structural integrity. Thus, understanding the effects of spike mutations on the protein structure is essential to understand the emerging variant evolution and possibly predict future variants to monitor for.

In our previous study, 110 SARS-CoV-2 samples were obtained from patients at Kasr Al-Aini Hospital and the Children’s Cancer Hospital Egypt 57357 between May 2020 and January 2021, covering the first two waves of the pandemic. Total RNA sequencing was used to sequence the viral genomes, and the isolates identified belonged to Nextclades 19A, 19B, 20A, 20B and 20D, with no detected VOCs (Jalal et al., 2022). In this study, sample collection was extended to include the third and fourth waves of the pandemic, from March to December 2021, from Egypt Center for Research and Regenerative Medicine (ECRRM), and Children’s Cancer Hospital Egypt (CCHE 57357). We used amplicon-based sequencing which provides greater depth of coverage over total RNA sequencing, resulting in higher sensitivity and better-quality sequencing data for mutation detection and phylogenetic analysis. Several variants appear in this study, including the VOCs, alpha, delta, and omicron. We identified mutations and mutation patterns throughout the whole genome, and identified several groups of co-occurring mutations. Finally, we investigated the effect of spike mutations, single or combined, on the spike protein thermodynamic stability, in an attempt to understand how and why the SARS-CoV-2 evolves.

## Materials and methods

### Ethical approval

CCHE 57357 Scientific and Medical Advisory Committee (SMAC) approved all experimental protocols used in this study. All processes that utilized human subjects had been performed by the institutional research committee’s ethical standards, as well as the 1964 Declaration of Helsinki and its later amendments or comparable ethical standards. Every patient agreed to sign valid consent as their willingness to participate in the present research.

### Sample collection and RNA extraction

Nasal/pharyngeal swabs were taken from 320 patients, 82 from ECRRM, and 238 from CCHE 57357 in viral transport medium. RNA was extracted using QIAamp^Ⓡ^ Viral RNA Mini kit (Qiagen). Confirmatory qualitative commercial RT-PCR kits were used for diagnosis and screening (depending on availability).

### Library preparation and next-generation sequencing

Library preparation was performed using AviSeq™ COV19 NGS Library prep kit from Avicenna™ (South Croydon, United Kingdom). Samples were then normalized, pooled and subjected to 150-base paired-end sequencing using Illumina MiSeqDx system with a minimum of 350 Mb sequencing depth per sample.

### Bioinformatics analysis

#### Quality control

**Figure 1:**
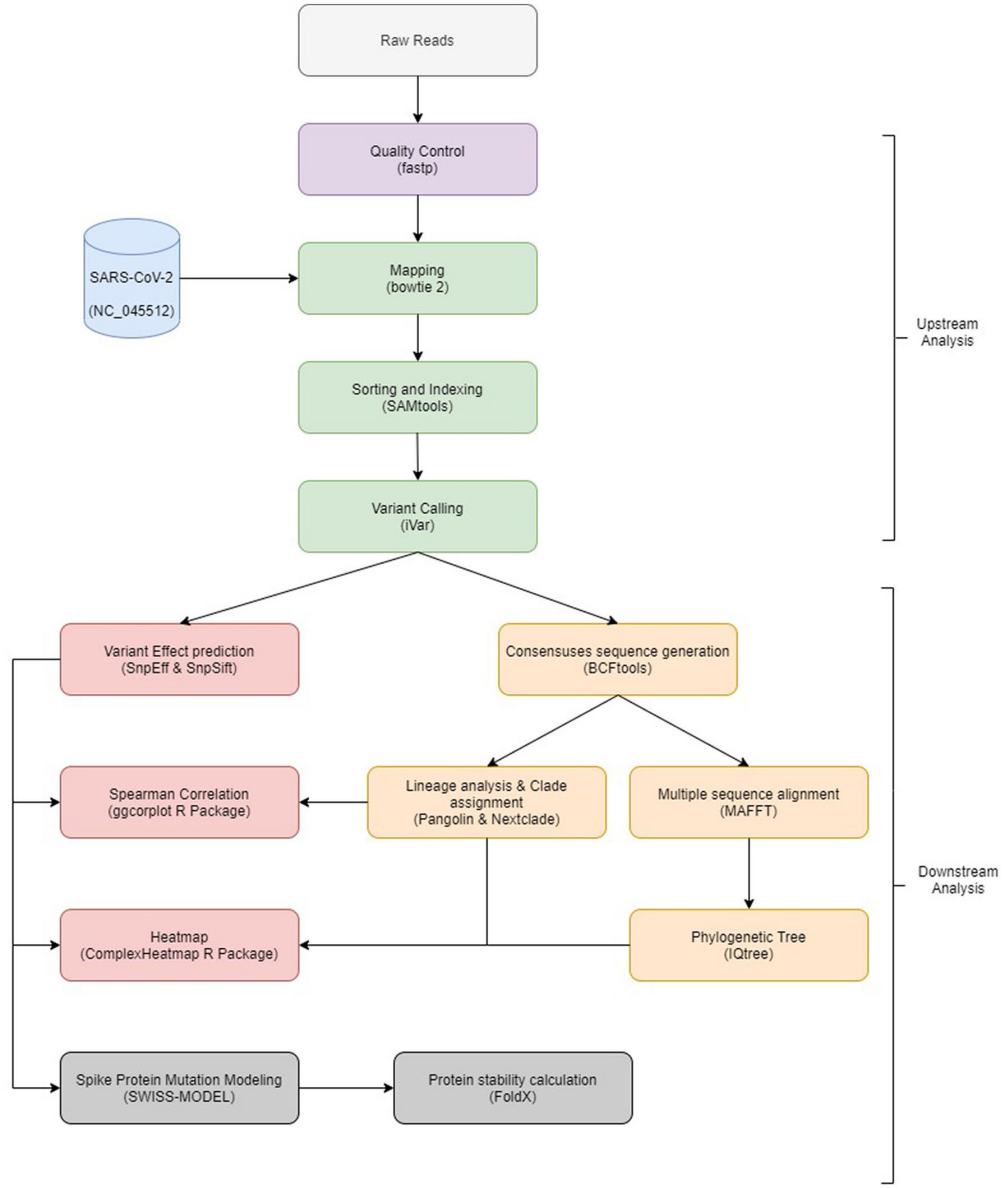
Overview of the bioinformatics workflow. Each box represents a task in the upstream and downstream analysis, with the major bioinformatics tools employed mentioned.

The bioinformatics analysis workflow is summarized in Figure 1. Initial quality control inspection of raw reads was done using FastQC (Andrews, 2010) and low-quality reads were trimmed using fastp (Chen et al., 2018).

#### Mapping and variant calling

Filtered reads were mapped to SARS-CoV-2 Wuhan-Hu-1 (MN908947.3) sequence using Bowtie 2 (Langmead and Salzberg, 2012), followed by sorting and indexing of mapped reads using SAMtools (Li et al., 2009). Then, iVar (Grubaugh et al., 2019) was used to remove primer sequences as well as variant calling which was followed by variant annotation and variant functional effect prediction using SnpEff (Cingolani et al., 2012) and SnpSift (Cingolani et al., 2012). Consensus sequences were generated using BCFtools (Danecek et al., 2021). Generated sequences were submitted to Pangolin (O’Toole et al., 2021) and Nextclade (Aksamentov et al., 2021) to perform lineage analysis and clade assignment.

#### Sequence analysis

Multiple sequence alignment and phylogenetic analysis were performed for all consensus sequences using MAFFT (Katoh et al., 2002) with options for rapid calculation of full-length MSA of closely related viral genomes feature (−-6merpair and –addfragments options) and IQtree (Minh et al., 2020) with 1,000 bootstrapping. The best fit model was GTR + F + R2 tested by IQtree. R package ggtree (Yu et al., 2017) was used to visualize and annotate generated tree.

#### Variant analysis

Raw variants were filtered to exclude synonymous mutations and variants with occurrence in only a single sample. A heatmap was created to visualize filtered variants using R package ComplexHeatmap (Gu et al., 2016). Identified mutations were compared to the clade/subclade defining mutations listed in^1^ and unique/less reported mutations in each clade/variant were identified.

To analyses the mutations that occur together, and others that do not occur together, Spearman correlation was performed using cor function inside R V.4.2.2 (R Core Team, 2020). Correlations were considered positive if *r* > 0.5 and considered negative if *r* < −0.5 and significant if *p* value < 0.05. ggcorrplot (Taiyun Wei et al., 2021) package was used to visualize generated correlations.

#### Protein stability analysis

The 7KRQ PDB structure (Zhang et al., 2021) of the closed conformation of the SARS-CoV-2 spike protein D614G mutant was retrieved from the RCSB PDB database (Berman et al., 2000) along with its amino acid sequence in FASTA format. The string object of the amino acid sequence was manipulated using Python scripting to mutate the regions of interest, and the mutated sequences were exported as FASTA files. The SWISS-MODEL Modeling API (Guex et al., 2009; Bienert et al., 2017; Waterhouse et al., 2018) was used to build homology models of the mutated FASTA files, as well as the 7KRQ original FASTA file, using the 3D structure of the 7KRQ PDB entry as a template, to restrict the conformation of the output models to the closed conformation. Quality of generated models were inspected by QMEAN scoring function^2^ (Benkert et al., 2011). All the generated homology models were then submitted to a pyFoldX python script (Schymkowitz et al., 2005; Radusky and Serrano, 2022) to optimize their side chain coordinates and evaluate their stability. The energy tables generated by the pyFoldX script were exported as CSV files, and the total energies of the models were used to compare their thermodynamic stability numerically. The ΔG of the 7KRQ was used as the baseline, and ΔΔG values were calculated by the difference in ΔG between models having individual or combined mutations. A positive ΔΔG indicates a destabilizing mutation, whereas a negative ΔΔG indicates a stabilizing one. The 3D structure visualization of the protein models throughout the whole study was performed using The PyMOL Molecular Graphics System, Version 2.5.2 Schrödinger, LLC.

## Results

### Domination of delta variant and appearance of omicron early in the fourth wave

**Figure 2:**
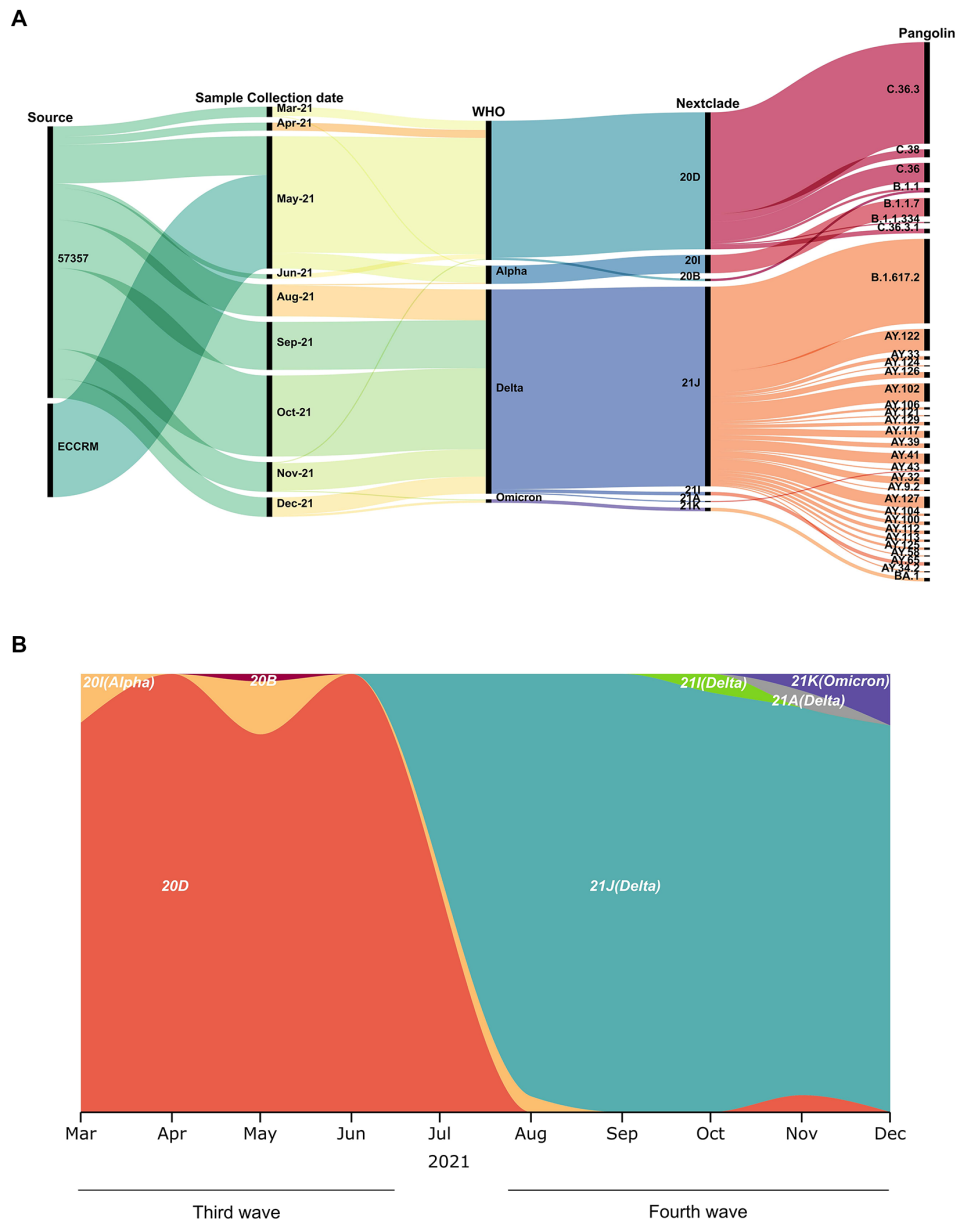
Sample collection date, source and variants identified. **(A)** Demographic, virologic, and sample collection data are shown in a multi-categorical alluvial diagram. **(B)** SARS-CoV-2 clade distribution over the ten-month study period from March to December 2021.

A total of 320 diagnosed positive COVID-19 samples were included in this study, spanning March to December 2021 (Figures 2A,B). Based on the wave pattern observed from the WHO data for COVID cases in Egypt [Egypt: WHO Coronavirus Disease (COVID-19), n.d.], we classified March to June as the third wave (136 samples) and August to December as the fourth wave (184 samples). Samples were selected randomly from the CCHE 57357 and ECRRM to cover the whole third and fourth waves. The alpha variant (Nextclade 20I, PANGO B.1.1.7) appears from March to May in 15 samples (11% of third wave samples), whereas the predominant variant in the third wave is Nextclade 20D (119 samples, 87.5% of third wave samples). The delta variant (Nextclades 21A, 21I and 21 J) appeared starting August 2021, in 179 samples (97% of fourth wave samples). The omicron (Nextclade 21 K, PANGO BA.1) variant, on the other hand, appeared in November and December in only three samples (1.6% of fourth wave samples).

### Omicron samples are phylogenetically closest to early pandemic variants

**Figure 3:**
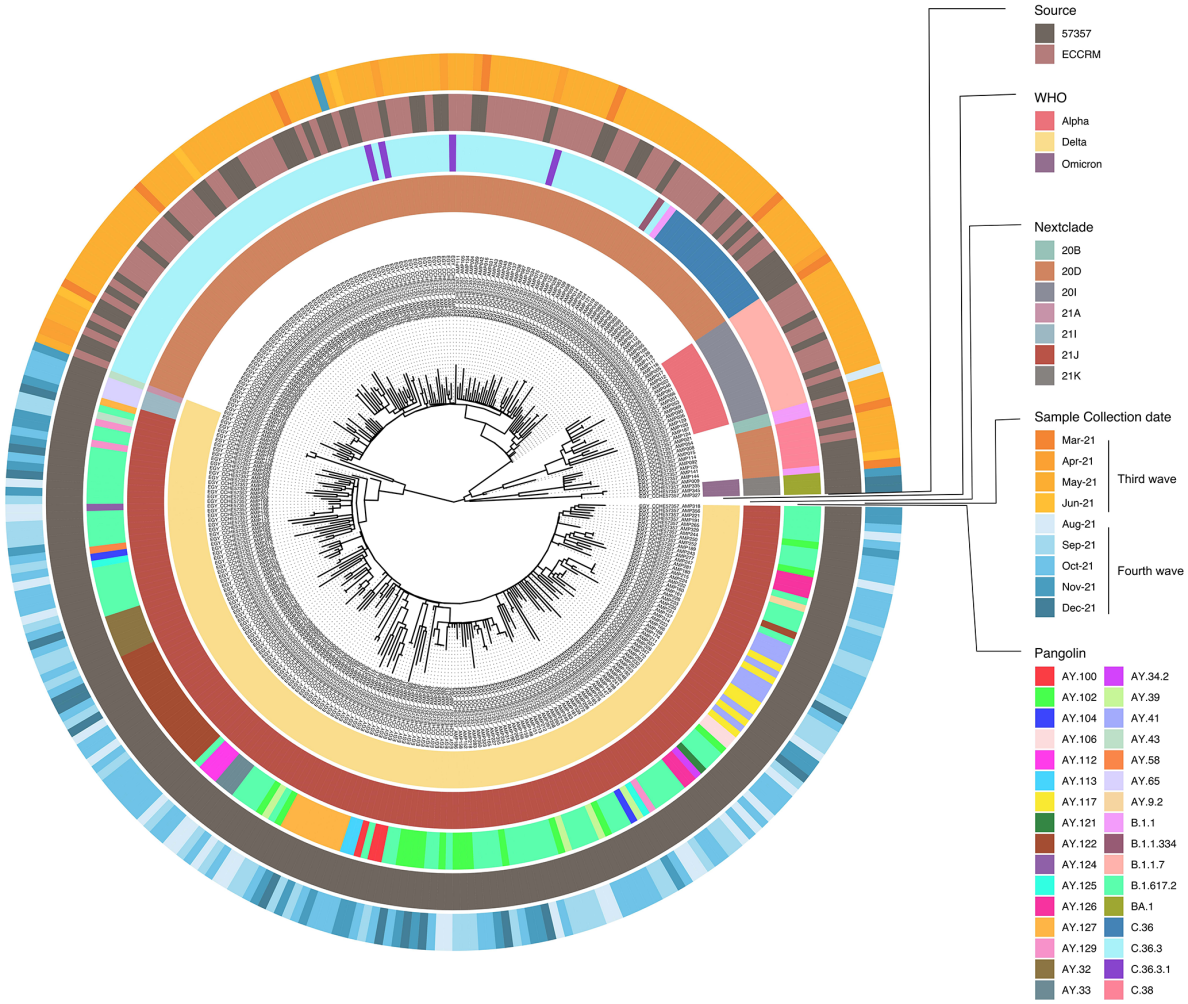
Phylogenetic analysis of 320 SARS-CoV-2 samples included in this study. Maximum likelihood phylogenetic tree of 320 SARS-CoV-2 sequences. Visualization of the tree was done using ggtree R package (Yu et al., 2017). For each sample; source of sample, date of sample collection, Nextclade, PANGO lineage and WHO VOC assignment are indicated by the circular color strip around the tree according to the legend.

To determine phylogenetic characteristics of our SARS-CoV-2 isolates, multiple sequence alignment of the full-length sequences was performed followed by maximum likelihood tree using IQ-tree and Nextstrain-based phylogenetic placements (Figure 3). Source, Nextclade, PANGO lineage, WHO VOC label, and date of sampling, are color indicated on the phylogenetic tree in Figure 3. The 320 SARS-CoV-2 sequences in this study belonged to 32 different PANGO lineages, 7 Nextclades, and 3 WHO VOCs. We did not observe a different phylogenetic pattern between samples obtained from ECRRM and CCHE 57357, indicating similar distribution of variants between both centers. We, however, observed a very different genomic sequence pattern between the third wave and the fourth wave, highlighted by the domination of the fourth wave by the delta variant. The three omicron samples group separately, and interestingly, are closest phylogenetically to Nextclades 20B and 20D which were found earlier in the pandemic.

### Unique mutation profiles in omicron samples across the whole viral genome

**Figure 4:**
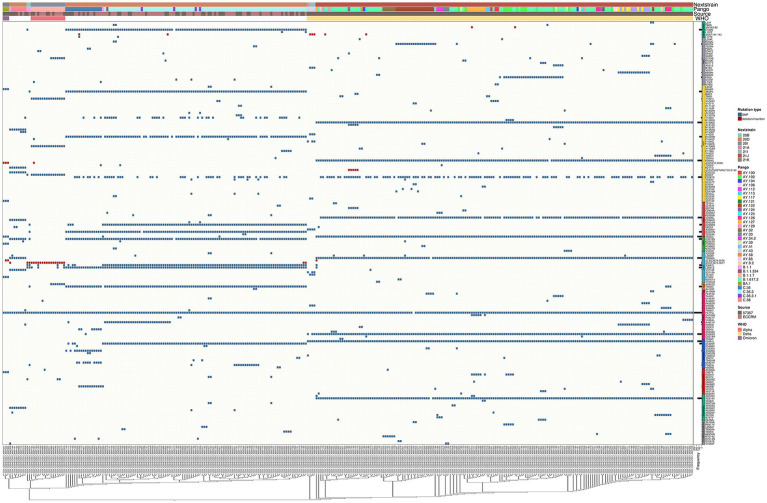
Complex heatmap showing mutations in ORF1ab across all samples. Type of mutation is indicated by different color; blue for SNPs and red for deletion/insertion mutations. PANGO lineage, Nextclade, Source of sample and WHO VOC assignment are color annotated as shown in legend.

**Figure 5:**
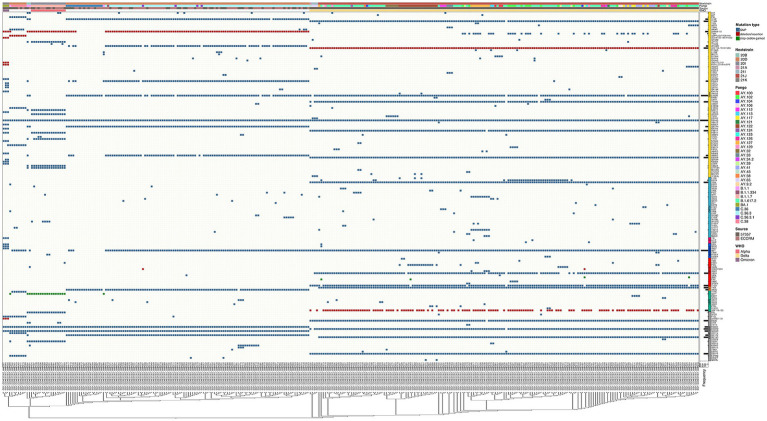
Complex heatmap containing mutations in other genes in SARS-CoV-2 across all samples. Type of mutation is indicated by different colors; blue for SNPs, red for deletion/insertion mutations, green for stop codon gained mutations. PANGO lineage, Nextclade, Source of sample and WHO VOC assignment are color annotated as shown in legend.

**Figure 6:**
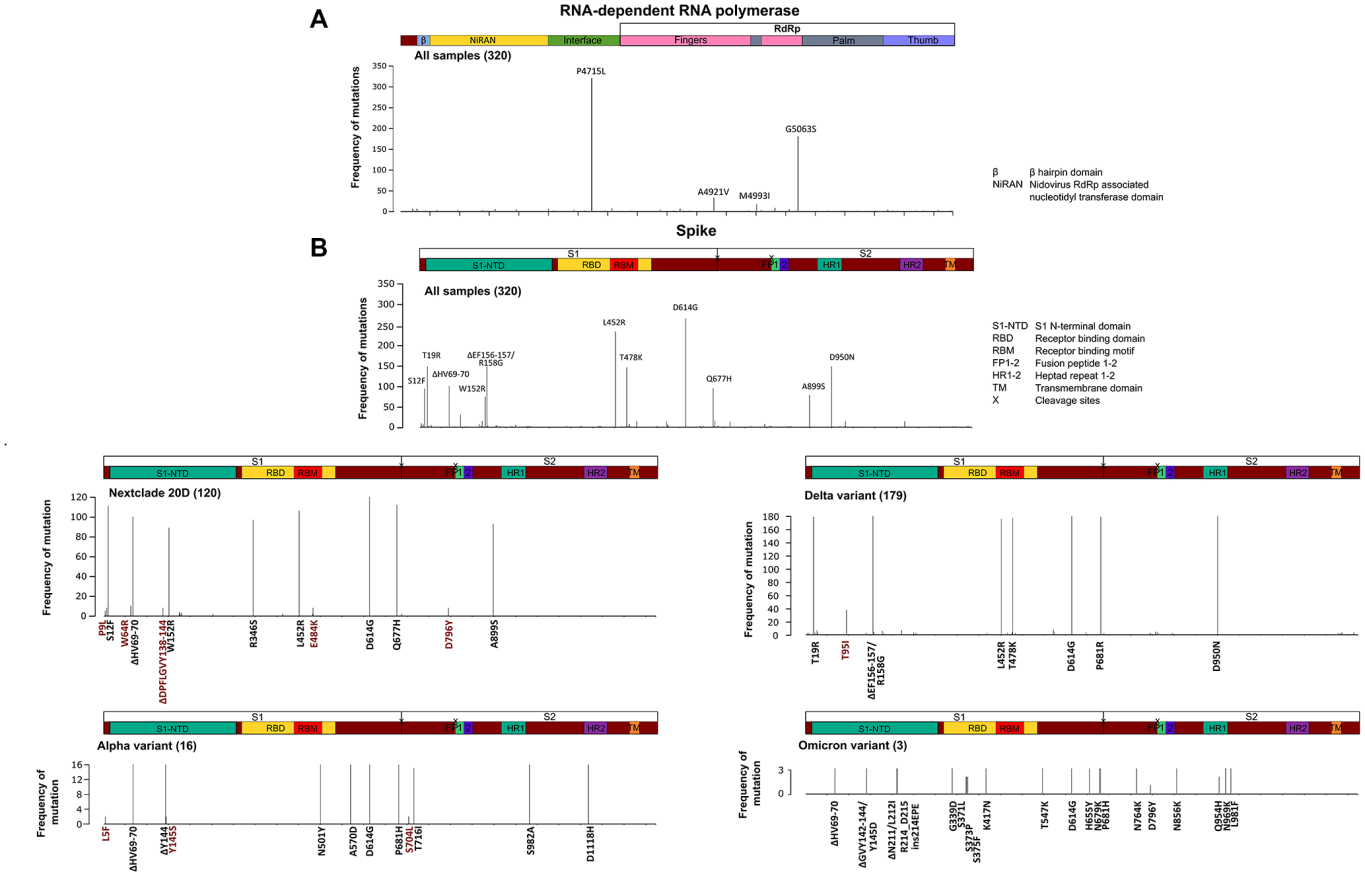
Structural domains, mutation positions and frequencies of SARS-COV-2 RNA dependent RNA polymerase and spike protein. A schematic diagram illustrating the domain arrangement of the **(A)** RNA dependent RNA polymerase and **(B)** spike protein. Mutations with frequencies >2 present across all samples, and in the main variants—Nextclade 20D, alpha, delta, and omicron—are shown. Mutations are labeled if they occur in more than 5% of variants and are color coded as follows: in black are clade/subclade defining mutations, and in red are mutations that are less/not reported in this variant.

The SARS-CoV-2 sequences in our samples were diverse, and comprised sequences from several Nextclade and PANGO lineages. Mutation analysis was performed compared to the Wuhan-hu-1 strain (MN908947.3), and mutation patterns were governed by Nextclade and PANGO lineages (Figures 4, 5). Detected nonsynonymous mutations in ORF1ab across the samples are shown in Figure 4, with the different peptides annotated. Nonsynonymous mutations in S, N, E, M, ORF3a, ORF7a-b and ORF8 are shown in Figure 5, with different color annotation for each gene. P4715L mutation in ORF1ab (RdRp), and D614G in the spike, are present in almost all the samples (Figures 4–6). Gain of stop codon mutations were observed in alpha variants at position 27 in ORF8, and in a few delta variants at position 90 and 95 in ORF7a. Mutations observed in RdRp and S, and the domains in which they occur are shown in Figures 6A,B respectively. The highly prevalent D614G occurs in S1, away from the receptor binding domain, whereas L452R and T478K which were found in 283 and 178 samples, respectively, occur within the receptor binding motif (RBM) in the RBD. Two deletion/insertion mutations, ΔHV69-V70 and ΔEF156-157/R158G, occur at the N-terminal domain of S1 in 121 (Nextclades 20D and 20I) and 179 (Delta variant) samples, respectively. We analyzed the spike mutations observed in different variants separately to identify common mutations versus new/less reported mutations. In addition to the commonly observed mutations in Nextclade 20D, we observed the mutations P9L, W64R, ∆DPFLGVY138-144, E484K, and D796Y. In the alpha variant, we observed three additional mutations: L5F, Y145S, and S740L. In the delta variant; however, we only reported one mutation besides the clade defining ones; T95I. A large amount of spike mutations appears in the omicron variant spanning most of the spike protein length. Five mutations occur in the S1 N-terminal domain: ΔH69-V70, ΔEF156-157, R158G, ΔN211, and L212I. Six mutations occur in the RBD: G339D, S371L, S373P, S375F, K417N, and T547K and several other mutations occur in the rest of the spike protein. Twelve omicron spike mutations were not observed in our samples, A67V, T95I, N440K, G446S, S477N, T478K, E484A, Q493R, G496S, Q498R, N501Y, and Y505H, possibly because of PCR failure in the amplicons covering this region during amplicon sequencing using AviSeq™ (Supplementary Figure 1).

### Several groups of co-occurring mutations appear with some negatively correlated mutations

**Figure 7:**
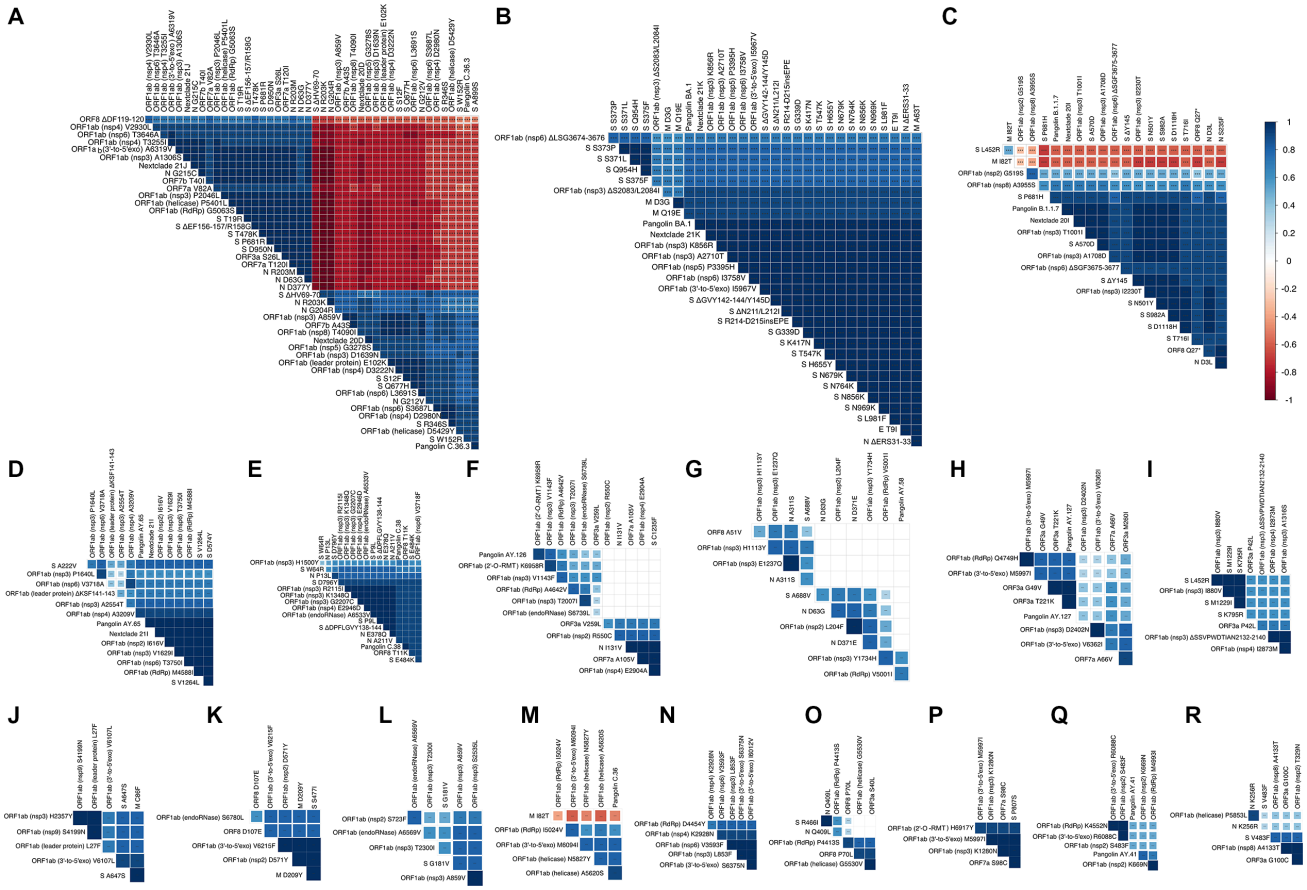
Correlation matrix representing showing highly correlated mutations and variants. In the correlation matrices, squares are sized and color-coded according to the magnitude of the correlation coefficient (r). The color code of r values is shown to the right (blue colors represent positive, red colors represent negative correlations between two parameters). Asterisks indicate statistically significant correlations (***p value < 0.005). Correlation analysis was generated by corrplot using nonparametric Spearman rank tests. Highly correlated mutations and variants are grouped and shown in panels **(A–R)**.

Eighteen groups of highly correlated mutations and variants were found by Spearman’s correlation coefficient and are shown in Figure 7. The first group and second groups occur in Delta variant (Nextclade 21J) and Nextclade 20D/PANGO C.36.3, respectively (Figure 7A). While showing very strong positive correlation within each of the groups, mutations in each group show strong negative correlation with mutations in the other group (*p* value < 0.001). Omicron variant (Nextclade 21 K, PANGO BA.1) showed a large amount of mutations in spike and other ORFs and are shown in Figure 7B. Highly correlated group of mutations observed in the Alpha variant (Nextclade 20I/PANGO B.1.1.7) are shown in Figure 7C, that were negatively correlated with L452R in spike, and I82T in membrane protein. Other groups of highly correlated mutations are shown in Figures 7C–R and Supplementary Figure 2.

### Stabilizing and destabilizing spike mutations explain their negative and positive correlations

**Figure 8:**
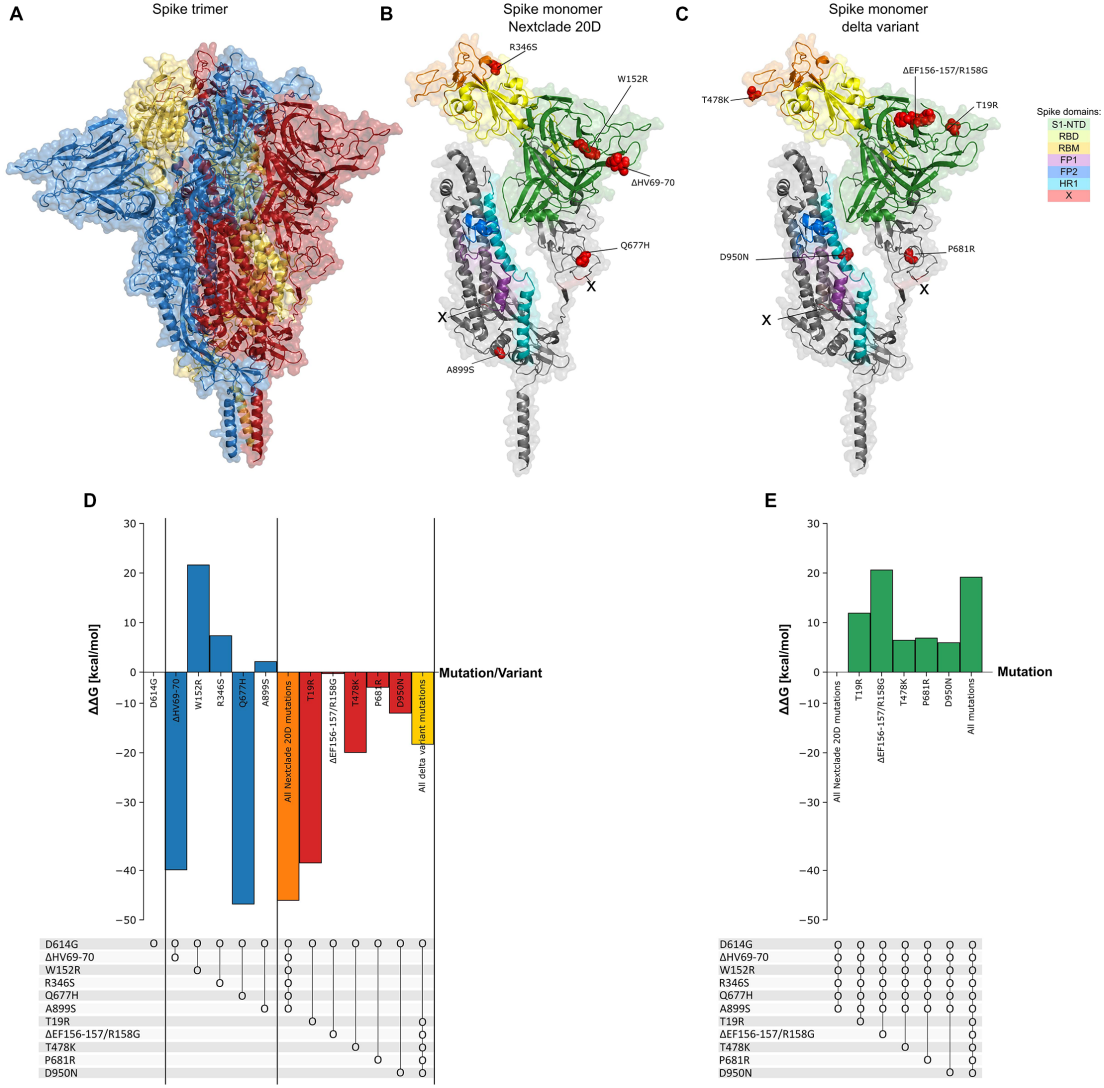
Spike protein thermodynamic stability analysis with mutations. **(A)** Structure of the spike protein in trimer form (PDB: 7KRQ). **(B,C)** Structure of monomeric spike showing its different domains with the mutations observed in Nextclade 20D and delta variants. **(D)** The effect of introduction of mutations (ΔΔG) observed in Nextclade 20D and delta variants, into spike containing only D614G mutation, individually and in combination on its folding Gibbs free energy (ΔG) (kcal/mol). ΔG of spike protein containing only D614G is used as baseline. **(E)** The effect of introduction of mutations (ΔΔG) observed in the delta variant, into spike protein containing all Nextclade 20D mutations, individually and in combination, on its folding Gibbs free energy (ΔG) (kcal/mol). ΔG of spike protein containing all Nextclade 20D mutations is used as baseline. Dot and line represent presence of combined mutations.

We find it intriguing that despite the high rate of mutations observed in spike protein across all SARS-CoV-2 variants, the mutations observed in Nextclade 20D (ΔHV69-70, A899S, R346S, Q677H, and W152R) do not co-occur with mutations observed in delta variant (S12F, T19R, ΔEF156-157/R158G, T478K, P681R and D950N) (*R* = −0.5–0.9, *p* value < 0.001, Figure 7A). We hypothesized that one reason to explain this was a negative effect on spike protein thermodynamic stability prohibiting their co-occurrence. To investigate this, we studied the effect of these spike mutations, individually and in combination, on the protein structure using SWISS-MODEL (Figures 8A–C) and calculated their impact on its thermodynamic stability (ΔG) using FoldX (Figures 8D,E). The ΔG of the spike protein with D614G mutation was used as the baseline, and ΔΔG values were calculated by the difference in ΔG between models having individual or combined mutations as indicated in Figure 8. A positive ΔΔG indicates a destabilizing mutation, whereas a negative ΔΔG indicates a stabilizing one. As seen in Figure 8D, combining the mutations observed in Nextclade 20D (ΔHV69-70, A899S, R346S, Q677H, and W152R) result in a more stable protein structure (ΔΔG = −46.066 kcal/mol). The effect of mutation S12F which is observed in Nextclade 20D was not tested as the spike template 7KRQ starts at amino acid residue 14, so preceding amino acids would not be accurately modeled. Similarly, combining the mutations observed in delta variant (T19R, ΔEF156-157/R158G, T478K, P681R, and D950N) also result in a more stable protein structure (ΔΔG = −14.564 kcal/mol) (Figure 8D). To understand why although many of the spike mutations in different variants are shared, mutations in Nextclade 20D and in delta variants have a very high negative correlation as seen in Figure 7A, we introduced the delta variant mutations in Nextclade 20D spike protein background individually and in combination and observed their ΔΔG values. Introduction of T19R, ΔEF156-157/R158G, T478K, P681R, and D950N in a spike containing all Nextclade 20D mutations results in ΔΔG values of 11.9, 20.62, 6.42, 6.87, and 5.93 kcal/mol, respectively, whereas introduction of all of these mutations together results in ΔΔG of 19.17 kcal/mol (Figure 8E). The less stable protein structure observed when the delta variant mutations are introduced with the Nextclade 20D mutations possibly explains why they do not occur together in SARS-CoV-2.

## Discussion

SARS-CoV-2 mutations have been studied and tracked since the spark of the pandemic in January 2020 with a major aim of new variant identification and classification, early prediction of further mutations, and clinical correlations with symptoms. Since the pandemic, SARS CoV-2 variants have been grouped by Nextclade into clades, PANGO lineage into lineages and sub lineages, and denoted by the WHO ‘variant of concern’ if a new variant shows high transmissibility or infectivity. The first identified VOC, the alpha variant initially appeared in England, and was estimated as 40–80% more transmissible than preceding variants. Despite contributing to the majority of the COVID-19 infections during the second and third waves in Europe, United Kingdom, and United States (Outbreak.Info SARS-CoV-2 data explorer [Internet], n.d.), we did not report any in our previous study spanning the first and second waves (Jalal et al., 2022), and only reported a few number of cases in this study covering the third and fourth waves of the pandemic. This is consistent with other studies showing the low spread of the alpha variant in Egypt (Roshdy et al., 2022; Seadawy et al., 2022) and Africa (He et al., 2021; Wilkinson et al., 2021; Tegally et al., 2022) during the same time period. While Nextclade 20D dominated the third wave, the fourth wave was dominated by the delta variant in our study and Africa (Wilkinson et al., 2021; Tegally et al., 2022). We also report three omicron samples representing the beginning of the omicron spread that would later dominate the COVID-19 pandemic.

The omicron variant accumulated over 50 mutations, including 32 in the spike protein alone (Saxena et al., 2022). Phylogenetic analysis of our samples shows the grouping of the omicron variants closest to early pandemic variants, 20B and 20D (Figure 3). Several studies have concluded that the omicron did not emerge from other VOCs, including the delta variant (Dhawan et al., 2022; Jung et al., 2022). How the omicron accumulated this large number of mutations in a short period of time, and from where it originated is still a subject of debate. Some studies suggest the omicron variant developed in an animal and returned back to humans (Wei et al., 2021; Jung et al., 2022). Other studies suggest the development in an immunocompromised patient, or in a subset of the population then re-introduced to the public (Aydillo et al., 2020; Choi et al., 2020). Of the 32 spike mutations, only 20 were detected in our samples, this is due to amplicon PCR failure during the viral genome sequencing, indicating the unsuitability of AviSeq™ library preparation kit for sequencing of omicron variants.

While most mutations that arise in the SARS-CoV-2 genome are synonymous and missense mutations, we report some stop codon gain mutations that result in a truncated protein. Q27* mutation observed in ORF8 results in a truncated version of only 26 amino acids, and has been observed predominantly in the alpha variant (Figure 5; Pereira, 2021a; DeRonde et al., 2022). While ORF8 is obviously not crucial for viral replication and survival, it has been implicated in immune response modulation and linked to improved viral transmissibility and less severe clinical picture (Pereira, 2021a,b). Stop codon gain in ORF7a was also observed in four of our samples at positions 90 and 95, also indicating it is not essential for viral replication and stability.

Throughout SARS-CoV-2 evolution and variant emergence, the spike protein remains the highest mutated region in the SARS-CoV-2 genome, in specific the S1 region (Figure 6B). S2 is highly conserved in SARS-CoV-2 and other CoVs, and is immunogenic upon infection (Voss et al., 2021; Ng et al., 2022), making it a promising candidate for vaccine development. Despite being argued to have limited effect on SARS-CoV-2 protection as they do not affect the binding of RBD to ACE2 receptor, potent neutralizing antibodies against S2 have been isolated (Chi et al., 2020; Pinto et al., 2021) and S2-targetted vaccines have shown promising efficacy (Ng et al., 2022).

An inevitable effect of viral evolution is the development of genome mutations, whether or not favorable for the viral fitness. Prevalence of a spike mutation or variant depends on the favorable effect this mutation has, alone or in combination, on ACE2 receptor binding (Ozono et al., 2021), glycosylation/cleavage (Tortorici and Veesler, 2019), and immune evasion (Harvey et al., 2021), as well as protein stability (Berger and Schaffitzel, 2020; Shorthouse and Hall, 2021). Interestingly, while generally some mutations in spike protein are shared between different variants (Magazine et al., 2022), we observed a strong negative correlation between two sets of highly correlated spike mutations in Nextclade 20D and delta variant (*p* value < 0.001) (Figure 7A). To investigate this, we utilized spike protein stability studies and observed a stabilizing effect of the combined Nextclade 20D mutations, and delta variant mutations. On the other hand, introduction of delta variant mutations, individual or combined, into Nextclade 20D spike protein background revealed a high destabilizing effect for each of the mutations, possibly explaining why they do not co-occur. Effects on spike protein stability may explain why some seemly unfavorable, or purposeless mutations prevail in a particular variant. Our results support a general consensus that virus evolution, particularly in the early pandemic, was strongly governed by a more stable spike protein. Later in the pandemic, following the selection force from development of and vast use of vaccines, the role of spike mutations in protein stabilization becomes less significant compared to mutations assisting in immune evasion, possibly explaining the high prevalence of some less stable spike protein omicron variants.

Altogether, our characterization of the SARS-CoV-2 genomes identified several variants and genome mutations. Based on our results, we do not recommend the use of AviSeq™ amplicon-based whole genome sequencing (WGS) for the detection of mutations in the currently dominating SARS-CoV-2 variant, omicron. We identified patterns of co-occurring mutations in the different ORFs of SARS-CoV-2 genome, as well as some negatively correlated mutations. Our results on the effects of spike protein mutations on protein stability show a general inclination toward a more stable protein structure, and partly explains the patterns of mutations observed in the spike protein. These findings hopefully help anticipate developing mutations for better management of the COVID-19 pandemic through more suitable vaccines and treatment plans.

## Data availability statement

The datasets presented in this study can be found in online repositories. The names of the repository/repositories and accession number(s) can be found at: NCBI - PRJNA905683.

## Ethics statement

The studies involving human participants were reviewed and approved by Children’s Cancer Hospital Egypt CCHE 57357. The patients/participants provided their written informed consent to participate in this study.

## Author contributions

AS contributed to the conception and design of the study. AS, KA, ME, WH, TM, SS, RH, GA-T, MH, and IA contributed to the sample collection and project facilitation. DJ, MGE, AD, and HE-S performed the sequencing experiments. OS and IM performed bioinformatics and statistical analysis. DJ and OS performed data analysis, interpretation, and figure generation. DJ wrote the first draft of the manuscript. DJ, LE, DH, and AM wrote sections of the manuscript. All authors contributed to manuscript revision and read and approved the submitted version.

## Funding

This project was funded by the Association of Friends of the National Cancer-free Initiative.

## Conflict of interest

The authors declare that the research was conducted in the absence of any commercial or financial relationships that could be construed as a potential conflict of interest.

## Publisher’s note

All claims expressed in this article are solely those of the authors and do not necessarily represent those of their affiliated organizations, or those of the publisher, the editors and the reviewers. Any product that may be evaluated in this article, or claim that may be made by its manufacturer, is not guaranteed or endorsed by the publisher.

## Footnotes

^1^ https://covariants.org/

^2^ https://pubmed.ncbi.nlm.nih.gov/21134891/
