# Supplementary material for: Genomic characterization of SARS-CoV-2 in Egypt: insights into spike protein thermodynamic stability
Source: Front Microbiol. 2023 Jun 2;14:1190133. doi: 10.3389/fmicb.2023.1190133 (PMC10273679; doi:10.3389/fmicb.2023.1190133)

Supplementary Figure 1: Amplicon coverage across the whole genome in twelve samples representing the four major clades in our samples.

IGV view for the amplicons in twelve isolates, three Nextclade 20D variants, three alpha variants, three delta variants and three omicron variants. The scale was fixed at 100 for comparison. (a) represents the coverage of different amplicons across the whole genome, (b) represents the coverage of different amplicons across the heavily mutated spike gene region

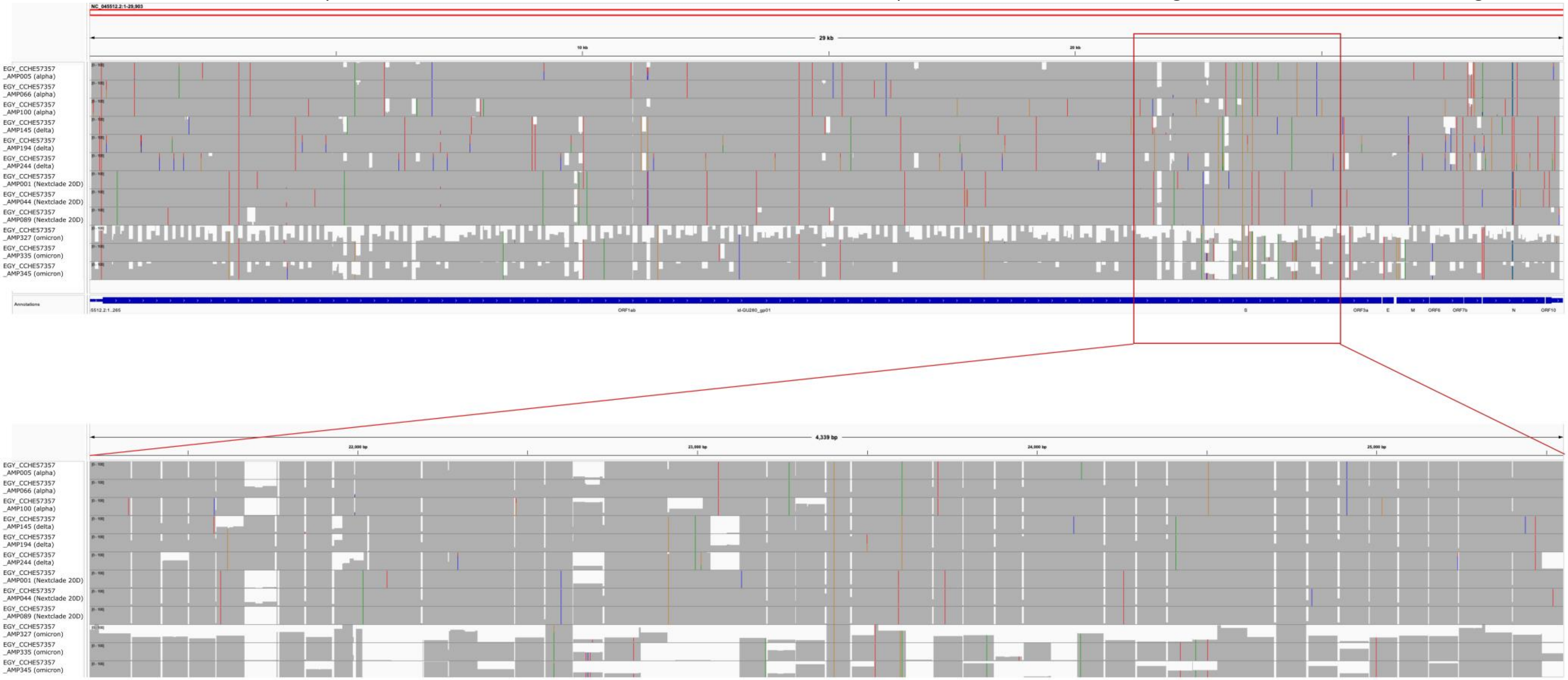

Supplement: Supplementary file 1 [file Image_1.pdf]
