# Supplementary material for: Genomic characterization of SARS-CoV-2 in Egypt: insights into spike protein thermodynamic stability
Source: Front Microbiol. 2023 Jun 2;14:1190133. doi: 10.3389/fmicb.2023.1190133 (PMC10273679; doi:10.3389/fmicb.2023.1190133)

**Supplementary Figure 2: Correlation matrix representing showing smaller groups of highly correlated mutations and variants.**

In the correlation matrices, squares are sized and colour-coded according to the magnitude of the correlation coefficient (r). The colour code of r values is shown to the right (blue colours represent positive, red colours represent negative correlations between two parameters). Correlation analysis was generated by corrplot using nonparametric Spearman rank tests. Asterisks indicate statistically significant correlations (****p*value < 0.005).

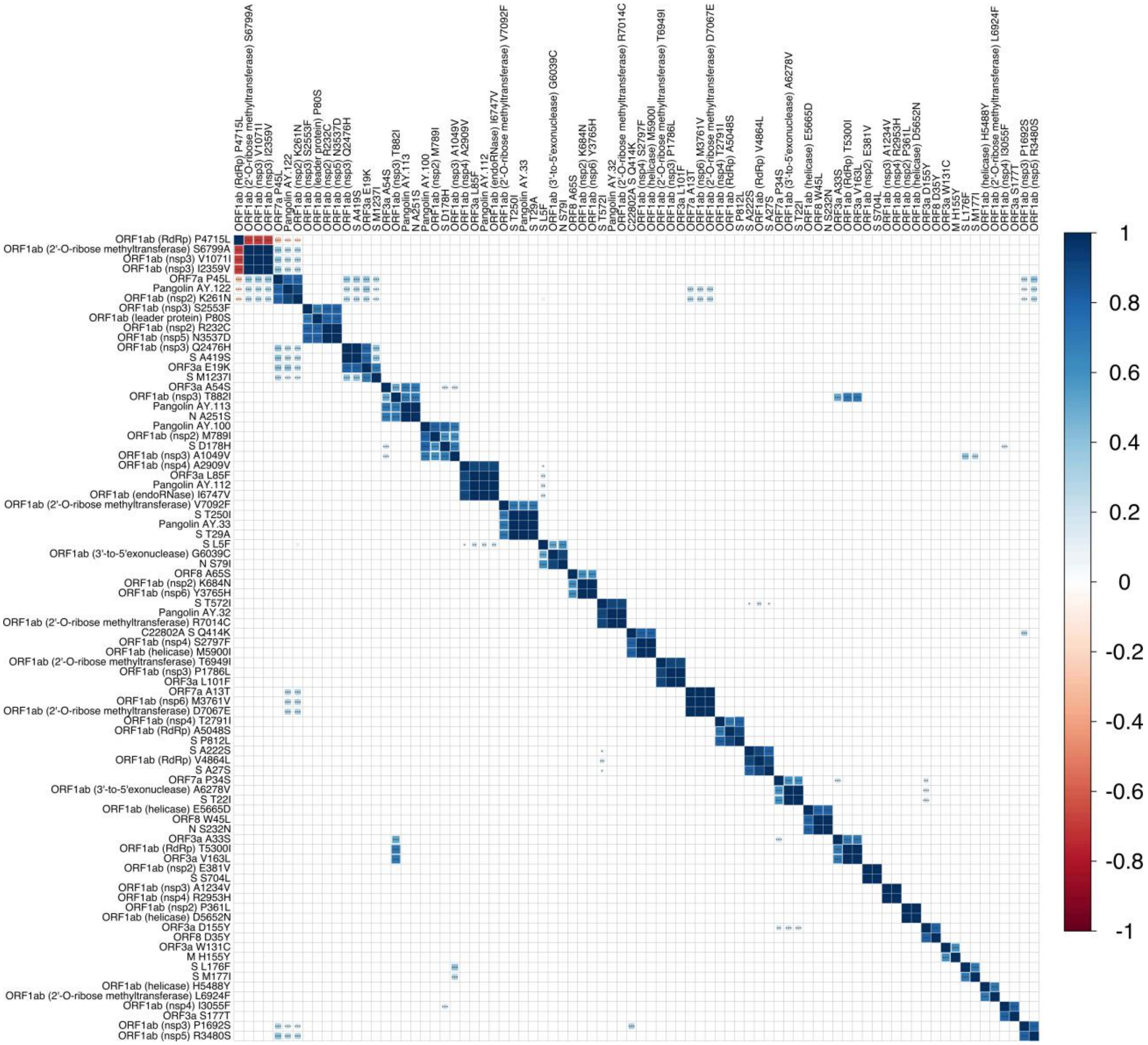

Supplement: Supplementary file 2 [file Image_2.pdf]
